# Post-COVID-19 physical and mental quality of life: a latent profile analysis and predictive factors

**DOI:** 10.1007/s11136-026-04206-y

**Published:** 2026-03-13

**Authors:** Marco Viola, Silvia Testa, Carlotta Sacerdote, Manolis Kogevinas, Eva Pagano, Rosalba Rosato

**Affiliations:** 1 Department of Psychology, University of Turin, Via Verdi 10, 10124 Turin, Italy; 2 Universitat Pompeu Fabra, Barcelona, Spain; 3 Barcelona Institute for Global Health, Barcelona, Spain; 4 Department of Human and Social Sciences, University of Valle d’Aosta, Aosta, Italy; 5 Unit of Cancer Epidemiology, Città Della Salute E Della Scienza, University-Hospital, Turin, Italy

**Keywords:** Post-COVID-19 conditions, Long-term, Latent profiles, Risk factors, Hospitalised patients

## Abstract

**Purpose:**

The long-term effects of SARS-CoV-2 infection are increasingly recognized, with heterogeneous physical and psychological symptoms that may persist for months, significantly affecting Health Related Quality of Life (HRQoL), functional capacity, and psychosocial well-being. This study explores distinct profiles of HRQoL and psychological symptoms in former COVID-19 inpatients and assesses the impact of clinical variables at admission on long-term outcomes.

**Methods:**

Patients hospitalised for COVID-19 at Molinette Hospital in Turin were contacted several months post-discharge (between June 2022 and June 2023) to complete a questionnaire assessing long-term HRQoL, sleep quality, depression, anxiety, stress, and fatigue. Clinical data at the time of hospitalisation were also available for each participant. A Latent Profile Analysis (LPA) was conducted on these physical and psychological variables, followed by multinomial logistic regression to examine how selected indicators of baseline COVID-19 severity and patient characteristics predicted profile membership.

**Results:**

The sample consisted of 601 patients. LPA identified three health-related profiles: *Fit-Vital* (n = 289), *Frail-Weak* (n = 229), and *Shattered-Broken* (n = 83). Neither ICU admission nor pneumonia showed significant associations with more severe physical and psychological conditions. Key predictors of membership in the more severe profiles included the presence of comorbidities and altered clinical parameters reflecting overall physical status. Female gender was identified as a risk factor for poorer outcomes.

**Conclusion:**

This study highlights a wide spectrum of post-COVID-19 conditions, ranging from good to severely compromised physical and mental health. Female gender, presence of comorbidities, and elevated early warning scores at hospital admission are risk factors for worse outcomes, emphasizing the need for comprehensive long-term care.

**Supplementary Information:**

The online version contains supplementary material available at 10.1007/s11136-026-04206-y.

## Abstract (unstructured)

The long-term impact of COVID-19 on both physical and mental health is an important issue. Many people continue to experience persistent symptoms months after infection, which can reduce their quality of life and overall well-being. However, the specific profiles of these long-term conditions in the Italian population have not been fully described.

In this study, we examined 601 patients who had been hospitalised for COVID-19 at Molinette Hospital in Turin. Several months after discharge, they completed questionnaires about their quality of life, sleep quality, depression, anxiety, stress, and fatigue. Using these data, we identified three distinct groups: those in good health (“Fit-Vital”), those with moderate difficulties (“Frail-Weak”), and those with severe ongoing problems (“Shattered-Broken”).

The severity of the initial infection, such as pneumonia or admission to intensive care, was not strongly linked to worse long-term outcomes. Instead, being female, having comorbidities, and showing poor health indicators at hospital admission were associated with belonging to the more vulnerable groups.These findings highlight the need for long-term care strategies that go beyond the acute phase of COVID-19. Paying attention to risk factors present at admission may help identify patients who require ongoing medical and psychological support.

**Supplementary Information:**

The online version contains supplementary material available at 10.1007/s11136-026-04206-y.

## Introduction

The long-term consequences of infection with Severe Acute Respiratory Syndrome Coronavirus 2 (SARS-CoV-2) are increasingly evident and remain highly variable and heterogeneous across individuals [1]. A growing body of research has documented the persistence of a range of physical and psychological symptoms, collectively referred to as post-COVID-19 condition, which can extend well beyond the resolution of acute illness [2–4]. These symptoms commonly include fatigue, muscle weakness, respiratory impairments, anxiety, depression, and cognitive disturbances, and they can significantly impair patients’ Health Related Quality of Life (HRQoL), functional capacity, and psychosocial well-being, sometimes persisting for months or even years after the acute phase of infection [5–7]. At the same time, several studies have shown that psychological responses to the COVID-19 pandemic have been far from uniform, with substantial individual differences in adaptation and recovery. Research conducted in community-based samples has revealed distinct mental health trajectories during and after the pandemic [8–10], with a significant proportion of individuals showing resilience or even positive psychological changes resulting from lifestyle adjustments imposed by the pandemic [11]. In other words, initial predictions of a widespread mental health crisis following the COVID-19 pandemic may have been somewhat overstated [12].

Given this variability, there is growing recognition of the need to investigate the long-term consequences of COVID-19 using person-centered approaches, which can capture heterogeneity across individuals rather than focusing on group averages. In particular, individuals who required hospitalization during the acute phase may represent a subgroup at increased risk for persistent physical and psychological complications [13, 14].

Identifying specific physical and psychological HRQoL profiles is a key area of investigation, with potential benefits for both risk stratification and long-term care. This approach would enable the development of more personalized follow-up strategies, particularly for individuals who are most vulnerable to persistent sequelae. However, distinguishing specific profiles is challenging, largely due to the interplay among the clinical severity of the acute phase, a high burden of comorbidities, pre-existing chronic conditions, and various sociodemographic factors such as age, sex, and educational level. Furthermore, the task is complicated by the inconsistency and, at times, the contradictory nature of the findings reported in the current literature regarding the association between these risk factors and long-term outcomes [1, 15–18].

Although traditional classification techniques, like cluster analysis, have been used in previous studies [1, 19, 20], Latent Profile Analysis (LPA) offers a more advanced and statistically grounded approach. LPA is a model-based statistical technique used to identify unobserved (latent) subgroups within heterogeneous populations based on continuous observed variables. Unlike traditional clustering methods, LPA does not require predefined cutoffs or assumptions of group membership, offering a data-driven approach to uncover underlying subtypes [21]. In medical and psychological research, Latent Class or Profile Analysis has been increasingly used to characterize symptom patterns and physical and psychological functioning, providing a more nuanced understanding of interindividual variability and identifying meaningful person-centered profiles of mental health and QoL [8–10, 22, 23]. Within this framework, the present application of LPA represents a more statistically rigorous and model-based approach to subgroup identification, compared to studies that have employed traditional clustering techniques.

This study had two primary objectives. First, it aimed to identify distinct health-related profiles among individuals previously hospitalised for COVID-19, using different late psychological information, regarding HRQoL, sleep quality, anxiety, depression, stress, and fatigue. Second, it examined whether baseline characteristics collected at hospital admission, indicators of acute disease severity, and demographic variables, were associated with the likelihood of membership in each identified profile.

## Methods

### Study population

1162 patients hospitalised with COVID-19 at Molinette Hospital in Turin (Italy) between March 2020 and June 2022 [24], as part of the study “Monitoring of Hospitalised Patients with COVID-19”, were considered for inclusion in a cross-sectional follow-up conducted from June 2022 and June 2023 to assess post-discharge HRQoL. Eligible participants were those aged 18 to 80 years. A total of 601 individuals took part in the assessment. Each participant completed the questionnaire once, at a variable time point following diagnosis. Participation was voluntary, and no participant received any form of compensation for taking part in the study.

At the time of completion, participants provided information on their current health status, covering physical and psychological domains, including HRQOL, anxiety, depression, stress, sleep disturbances, and fatigue. Sociodemographic data, including age (categorized into groups: 18–54, 55–64, 65–74, and 75–80 years), gender, educational level (with or without a high school diploma), and marital status, were also collected. In addition, the time elapsed (in months) between hospitalisation for COVID-19 and questionnaire completion was recorded. For detailed sample characteristics, refer to the original publication [25]. Clinical data collected during hospitalisation were used to derive indices of disease severity and comorbidity. Data on ICU admission were also recorded.

The research was conducted in accordance with the STROBE guidelines for observational studies, with the study clearly defined as cross-sectional in nature. Ethical approval was granted by the Ethics Committee of Molinette—Città della Salute e della Scienza Hospital in Turin (Italy). Informed consent was obtained from all participants, and all data were pseudonymised to protect participant confidentiality.

### Measures

#### Health-related quality of life

HRQoL was assessed using the validated Italian version of the Short Form-36 (SF-36) questionnaire, a widely used self-administered instrument designed to evaluate general health status across a broad range of physical and mental conditions [26, 27]. The Italian adaptation of the SF-36 has demonstrated high reliability and has been validated in the general population. The SF-36 captures both physical and mental health dimensions through eight multi-item scales addressing functional ability, subjective well-being, and overall health perception: physical functioning (PF, 10 items), role limitations due to physical problems (RP, 4 items), bodily pain (BP, 2 items), general health perceptions (GH, 5 items), vitality (VT, 4 items), social functioning (SF, 2 items), role limitations due to personal or emotional problems (RE, 3 items), and general mental health (MH, 5 items). Scores for each SF-36 domain were calculated by summing the corresponding item responses and transforming the totals to a 0–100 scale, where 0 represents the lowest and 100 the highest possible level of HRQoL.

#### Sleep quality

Sleep quality was assessed using the Pittsburgh Sleep Quality Index (PSQI), a self-report instrument designed to measure sleep quality and disturbances over the past month. The questionnaire consists of 19 items aggregated into seven component scores: subjective sleep quality, sleep latency, sleep duration, habitual sleep efficiency, sleep disturbances, use of sleep medication, and daytime dysfunction. Each component score ranges from 0 to 3, and their sum yields a global score ranging from 0 to 21, with higher scores indicating poorer sleep quality. A global PSQI score greater than 5 is commonly used to define poor sleep quality [28]. The PSQI demonstrates high test–retest reliability and good validity, including in patients with primary insomnia. This makes it a particularly valuable tool for assessing sleep quality in individuals whose insomnia is not secondary to other disorders [29].

#### Depression, anxiety, stress

To assess levels of depression, anxiety, and stress, the short-form version of the Depression Anxiety Stress Scales (DASS-21) was used [30, 31]. This instrument is a 21-item self-report questionnaire designed to measure the severity of symptoms commonly associated with these three psychological states. Participants are asked to rate the extent to which each item applied to them over the past week using a 4-point Likert scale ranging from 0 (never) to 3 (almost always). The scale consists of three subscales, depression, anxiety, and stress, each comprising 7 items. Subscale scores are calculated by summing the responses to the relevant items, resulting in three total scores, each ranging from 0 to 21. Higher scores indicate greater symptom severity in the corresponding domain. The severity cut-offs for each subscale are as follows: depression: 0–4 = normal; 5–6 = mild; 7–10 = moderate; 11–13 = severe; ≥ 14 = extremely severe,

anxiety: 0–3 = normal; 4–5 = mild; 6–7 = moderate; 8–9 = severe; ≥ 10 = extremely severe,

stress: 0–7 = normal; 8–9 = mild; 10–12 = moderate; 13–16 = severe; ≥ 17 = extremely severe [30].

The primary aim of the DASS-21 is to quantify the severity of fundamental emotional distress symptoms. As such, it serves both as a diagnostic aid in identifying levels of depression, anxiety, and stress, and as a tool for monitoring changes in symptom severity over the course of treatment or intervention [32].

#### Fatigue

The Fatigue Severity Scale (FSS-9) was utilized to assess fatigue severity [33, 34]. The FSS-9 is a unidimensional scale consisting of 9 items that capture information to assess the impact and severity of fatigue experienced over the past two weeks. Each item is rated on a 7-point Likert scale ranging from 1 (strongly disagree) to 7 (strongly agree); higher scores indicate more severe fatigue. The total score is calculated as the average of all item scores and ranges from 1 to 7, indicating low to very high levels of fatigue, respectively [34]. A mean score of 4.0 or above is generally considered indicative of clinically significant fatigue [33].

#### Previously collected and archived variables

Clinical information documented in clinical records at the time of hospital admission was used to derive indices of the illness severity and comorbidity burden. The National Early Warning Score (NEWS2) integrates multiple physiological indicators, including respiratory rate, oxygen saturation, systolic blood pressure, heart rate, level of consciousness or acute confusion, and body temperature, into a composite score weighted by clinical relevance [35, 36]. This score was dichotomised into low risk (score ≤ 4) and medium/high risk (score > 4) to reflect acute illness severity [25].

Comorbidity burden was assessed using the Charlson Comorbidity Index (CCI), categorized as 0 versus ≥ 1. In addition, admission to the intensive care unit (ICU) and the presence of pneumonia at the time of emergency department admission were also recoded.

### Statistical analyses

Descriptive statistics were computed for selected patient characteristics, using means and standard deviations for continuous variables, and frequencies and percentages for categorical ones. Missing data on SF-36, DASS-21, FSS-9 and PSQI scales were addressed through Multiple Imputation by Chained Equations (MICE), incorporating key covariates such as age, gender, educational level, comorbidity and severity of illness [37, 38]; five imputed datasets were generated. Convergence of the iterative imputation process was evaluated using trace plots, confirming stable estimates across iterations, and one dataset was subsequently selected for the analyses [37]. Prior research indicates that MICE yields more reliable results when the proportion of missing data in variables ranges from approximately 2.5% to 30% [39, 40]. The MICE procedure was implemented using the *mice* package in RStudio (2023.06.01 + 524 release).

Latent Profile Analysis (LPA) was applied to evaluate the presence of distinct groups of participants characterized by similar profiles within groups and different between groups. Subsequently, associations between relevant predictors and the identified profiles were examined in order to explore the relationships and potential patterns underlying the profile structure. The first phase was carried out using Mplus (version 7), while the second was conducted with RStudio (2023.06.01 + 524 release).

#### Latent profile analysis

Latent Profile Analysis (LPA) was employed to identify distinct profiles that best captured the heterogeneity in physical and mental health status following hospitalisation for COVID-19. The LPA included the following observed indicators: the eight HRQoL domain scores (PF, RP, BP, GH, VT, SF, RE, MH), as well as scores assessing sleep quality, anxiety, depression, stress, and fatigue. To ensure comparability across domains, all indicator scores were standardized into z-scores based on the sample distribution, thereby enabling their inclusion in multivariate statistical models. Model fit was evaluated by estimating solutions sequentially, starting with a single profile and incrementally increasing the number of profiles. Estimation ceased when issues of empirical under-identification or convergence emerged. Model selection was guided by relative fit indices, in line with Sorgente et al. [41].

Assessment of relative model fit involved both statistical tests and descriptive indicators. Among the descriptive metrics, five information criteria (IC) were examined: the Akaike Information Criterion (AIC), the Consistent Akaike Information Criterion (CAIC), the Approximate Weight of Evidence (AWE), the Bayesian Information Criterion (BIC) and the Sample-size Adjusted Bayesian Information Criterion (saBIC), where lower values indicate superior model fit. The BIC also functions as an estimate of the Schwarz Information Criterion (SIC) [42], which underlies the computation of two additional relative indices: the approximate Bayes Factor (BF) and the approximate correct model probability (cmP), both widely adopted following the work of Nagin [43]. These indices jointly offer a robust framework for evaluating the relative adequacy of competing latent profile solutions.

Inferential evaluation of relative model fit can be conducted using several statistical tests, including the Vuong-Lo-Mendell-Rubin Likelihood Ratio Test (VLMR-LRT) [44, 45], the adjusted Lo-Mendell-Rubin likelihood ratio test (adjusted LMR-LRT) [45], and the Bootstrap Likelihood Ratio Test (BLRT) [46, 47]. These tests compare a model with k-1 latent profiles to the model with k profiles. A statistically significant p-value suggests that the model with k profiles offers a significantly better fit than the model with k-1 profiles. Conversely, a non-significant result indicates that the k-profile model does not provide a meaningful improvement, and thus the more parsimonious k-1 profile solution is preferred.

After identifying the optimal model, the accuracy of individual classification into latent profiles must be assessed [48]. A widely used metric for this purpose is entropy (E_k_), where values approaching 1 suggest higher classification precision. When entropy values are high (typically above 0.80), indicating reliable class assignment, it is appropriate to relate latent profiles to external variables using either a two-step or a bias-adjusted three-step approach. However, simulation results by Bakk and Kuha [49] indicate that the two-step estimator tends to be more efficient, performing as well as or slightly better than the three-step method, while avoiding the additional estimation step required in the latter. Additional diagnostic indicators include class proportions (CP_k_ or π_k_), the modal class assignment proportions (mcaP_k_), average posterior probabilities (avePP_k_), and odds of correct classification (OCC_k_). According to established guidelines, classification quality is considered acceptable when the mcaP_k_ falls within the 95% confidence interval of π_k_, avePP_k_ exceeds .70, and OCC_k_ are above 5 [41, 48]. Finally, the choice of the most appropriate model was guided also by a qualitative evaluation of the extracted profiles. This involved considering the theoretical meaning of each profile and its alignment with previous findings in the literature, ensuring that the selected model was both empirically robust and conceptually sound.

#### Relationship between predictors and identified profiles

After identifying the optimal latent profile model, we saved the most likely class membership for each participant to determine their classification within the corresponding physical and mental health identified profile. A multinomial logistic regression was then conducted to examine the associations between predictor variables (clinical parameters at COVID-19 diagnosis and associated hospitalisation) and covariates, and the dependent variable representing profile membership.

## Results

The mean age of participants was 61.0 years (SD = 12.4; range: 18–80), and 62.4% of the sample were male. Comprehensive demographic and clinical information are available in the Supplementary Material and in Viola et al. [25].

A total of 112 (18.6%) questionnaires showed at least one missing data on the scales and were imputed. The MICE procedure was applied separately to each scale to account for the different measurement structures and distributions of the SF-36, DASS-21, FSS-9, and PSQI scales. This approach ensured model stability and consistency across scales, given their distinct scoring systems and missing data patterns. Missing data on the SF-36, DASS-21, and FSS-9 scales were imputed at the item level. In contrast, for the PSQI scale, imputation was performed on the total score only, as the presence of time-related variables (e.g., sleep and wake times) makes item-level imputation less reliable and more complex. Convergence of the imputation process was evaluated using trace plots, which indicated stable parameter estimates and satisfactory convergence across iterations. All scales exhibited good internal consistency, as indicated by McDonald’s omega values greater than 0.84 (see Table S1 in the Supplementary Material for McDonald’s omega values and detailed information on missing data for each scale).

### Identification of latent profiles

We evaluated six latent profile models, ranging from one to six profiles. The seven-profile solution was excluded due to the extremely small number of individuals in one of the identified profiles. As shown in Table 1, the three-, four-, and five-profile solutions yielded acceptable fit indices. Specifically, since the values of AIC, CAIC, AWE, BIC, and SABIC decreased with each additional profile, without any Bayes Factors exceeding 3 or cmP values above 0.10, model selection was primarily guided by the results of the VLMR-LRT, adjusted LMR-LRT, and BLRT. The three-profile model showed statistically significant VLMR-LRT and adjusted LMR-LRT tests (*p *< 0.005), while the four- and five-profile models, although associated with non-significant test results, were retained for further comparison given their proximity to the conventional threshold (*p* < 0.10).

**Table 1:** Relative model fit indices for six latent profile models

Model	AIC	CAIC	AWE	BIC	SABIC	SIC	BF	cmP	VLMR-LRT test	LMR-LRT test	BLRT test
1-profile	22,211.32	22,351.69	22,570.05	22,325.69	22,243.14	− 11,162.84	0.00	0.00			
2-profile	18,884.55	19,100.49	19,436.43	19,060.49	18,933.50	− 9530.24	0.00	0.00	*p* < 0.0001	*p* < 0.0001	*p* < 0.0001
3-profile	17,872.61	18,164.14	18,617.66	18,110.14	17,938.70	− 9055.07	0.00	0.00	***p* < 0.005**	***p* < 0.005**	*p* < 0.0001
4-profile	17,430.45	17,797.56	18,368.66	17,729.56	17,513.67	− 8864.78	0.00	0.00	***p* = 0.0781**	***p* = 0.0807**	*p* < 0.0001
5-profile	17,183.47	17,626.16	18,314.84	17,544.16	17,283.83	− 8772.08	0.00	0.00	***p* = 0.0919**	***p* = 0.0944**	*p* < 0.0001
6-profile	16,957.75	17,476.01	18,282.28	17,380.01	17,075.24	− 8690.01	0.00	0.00	*p* = 0.2816	*p* = 0.2859	*p* < 0.0001

AIC, Akaike Information Criterion; CAIC, Consistent AIC; AWE, Approximate Weight of Evidence Criterion; BIC, Bayesian Information Criterion; SABIC, Sample-size Adjusted BIC; SIC, Schwarz Information Criterion; BF, Bayesian Factor; cmP, approximate correct model probability; VLMR-LRT, Vuong-Lo-Mendell-Rubin Likelihood Ratio Test; LMR-LRT, Lo-Mendell-Rubin Likelihood Ratio Test; BLRT, Bootstrapped Likelihood Ratio Test. Bold values indicate the three models retained for further comparison

Based on these results, the three-, four-, and five-profile solutions were further examined using classification diagnostics (see Table 2). All three models met the established criteria for classification quality, indicating that each profile represented a meaningfully distinct profile. However, the three-profile model was ultimately retained, as it showed statistically significant improvements on the VLMR-LRT and LMR-LRT tests. In contrast, the four- and five-profile solutions did not identify any additional meaningful differentiation beyond what was already captured by the three-profile solution.

**Table 2:** Classification diagnostics for the three-, four- and five-profile models

Entropy (E)	Profile (N)	CP	mcaP	AvePP	OCC
0.929	Profile 1 (289)	0.477 (0.405, 0.542)	0.481	0.973	39.51
	Profile 2 (229)	0.382 (0.333, 0.435)	0.381	0.960	38.83
	Profile 3 (83)	0.141 (0.096, 0.199)	0.138	0.974	228.22
0.908	Profile 1 (153)	0.249 (0.210, 0.306)	0.255	0.946	52.84
	Profile 2 (197)	0.329 (0.261, 0.383)	0.328	0.932	27.95
	Profile 3 (49)	0.083 (0.051, 0.119)	0.082	0.979	515.06
	Profile 4 (202)	0.339 (0.267, 0.418)	0.336	0.961	48.05
0.934	Profile 1 (48)	0.079 (0.052, 0.115)	0.080	0.973	420.13
	Profile 2 (88)	0.149 (0.097, 0.235)	0.146	0.939	87.92
	Profile 3 (120)	0.196 (0.150, 0.239)	0.200	0.940	64.27
	Profile 4 (107)	0.180 (0.137, 0.234)	0.178	0.938	68.92
	Profile 5 (238)	0.395 (0.321, 0.454)	0.396	0.978	68.09

CP, class proportion; mcaP, modal class assignment proportion; avePP, average posterior probability; OCC, odds of correct classification

**Fig. 1:**
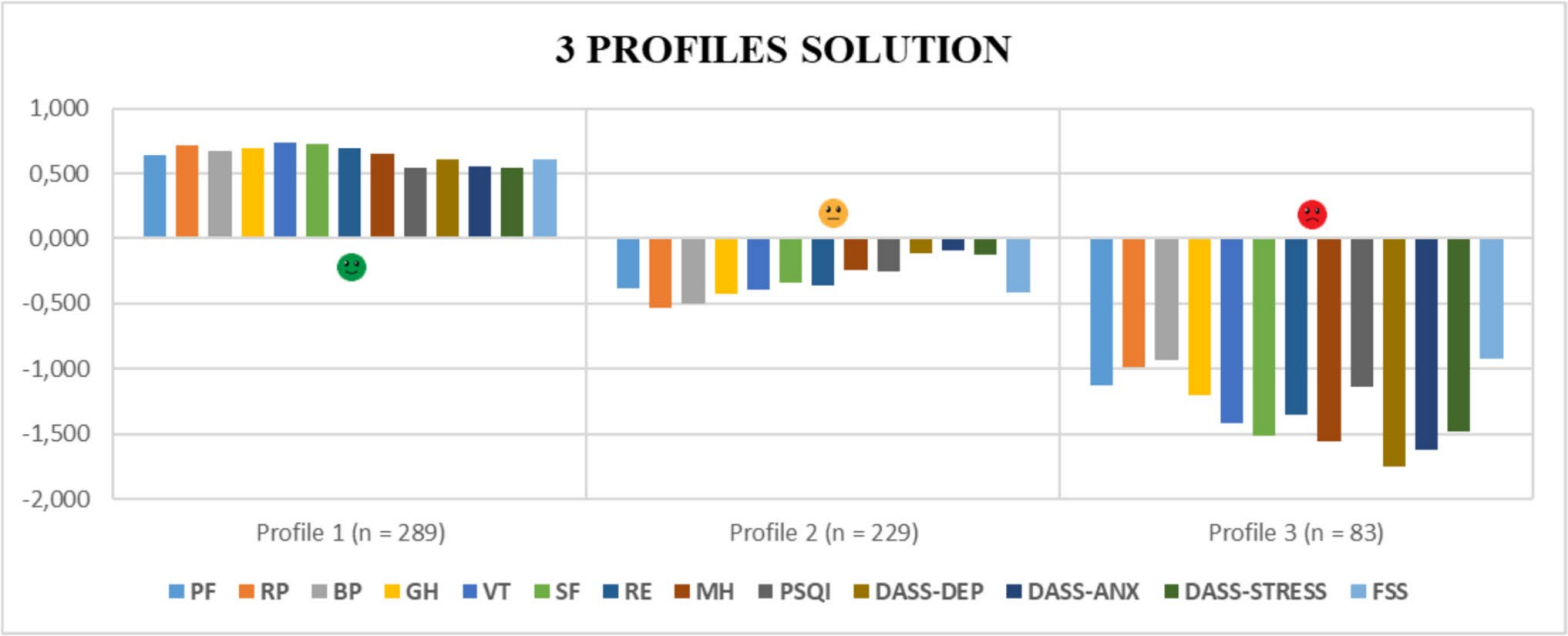
Representation of the three health-related health profiles with mean scale scores expressed as z-scores*. PF, physical functioning; RP, role limitations due to physical problems; BP, bodily pain; GH, general health perceptions; VT, vitality; SF, social functioning; RE, role limitations due to personal or emotional problems; MH, general mental health; PSQI, sleep score; DASS-DEP, depression score; DASS-ANX, anxiety score; DASS-STRESS, stress score; FSS, fatigue score. *For ease of interpretation, the scores of the PSQI, DASS-DEP, DASS-ANX, DASS-STRESS, and FSS scales were reversed in the figure only, so that higher (positive) values consistently indicate better conditions and lower (negative) values indicate poorer conditions across all scales

The three identified latent profiles (see Fig. 1), representing distinct patterns of mental and physical health, were labelled as follows: *Fit-Vital* (n = 289; 48.1%), *Frail-Weak* (n = 229; 38.1%), *Shattered-Broken* (n = 83; 13.8%).

As shown by the scale scores, both overall and across the three identified profiles (see Table 3), participants in Profile 1 (*Fit-Vital*, n = 289) reported good post-COVID-19 physical and mental health. This is evidenced by positive SF-36 scores on both physical and psychological dimensions, along with low levels of sleep disturbances, depression, anxiety, stress, and fatigue. Profile 2 (*Frail-Weak*), comprising 229 individuals, was characterized by slightly compromised physical and psychological functioning, particularly in the physical domain, as reflected in lower scores on role limitations due to physical problems (RP), vitality (VT), and higher scores on the FSS. Lastly, participants in the *Shattered-Broken* profile (n = 83, 13.8% of the sample) exhibited severely impaired physical and mental conditions, with consistently extreme scores, either markedly low on the SF-36 scales or markedly high on the symptom indicators.

**Table 3:** Means and standard deviations of scale scores, overall and across the three profiles

Scales	Overall Mean (sd)	Profiles		
		Fit-Vital Mean (sd)	Frail_Weak Mean (sd)	Shattered_Broken Mean (sd)
PF^1^	67.55 (29.57)	86.26 (16.98)	55.96 (26.38)	34.34 (26.91)
RP^1^	58.11 (42.03)	88.49 (22.52)	35.26 (36.25)	15.36 (30.45)
BP^1^	65.13 (28.69)	84.53 (20.05)	50.32 (21.24)	38.43 (26.43)
GH^1^	50.94 (22.98)	66.90 (16.24)	40.87 (16.21)	23.13 (15.63)
VT^1^	51.34 (21.52)	67.08 (13.89)	42.69 (12.83)	20.42 (14.23)
SF^1^	67.70 (25.54)	86.07 (13.45)	58.68 (17.02)	28.61 (19.18)
RE^1^	66.17 (41.16)	94.58 (15.88)	50.36 (39.57)	10.84 (22.76)
MH^1^	65.20 (20.59)	78.53 (12.30)	60.07 (14.47)	32.96 (15.39)
PSQI^2^	7.31 (4.36)	4.97 (3.01)	8.48 (3.84)	12.23 (4.31)
DASS-21–depression^3^	4.36 (4.22)	1.83 (1.85)	4.83 (3.04)	11.87 (3.54)
DASS-21–anxiety^3^	3.47 (3.59)	1.51 (1.66)	3.82 (2.47)	9.36 (4.32)
DASS-21–stress^3^	5.21 (4.19)	2.97 (2.66)	5.77 (3.11)	11.51 (4.23)
FSS-9^4^	4.01 (1.61)	3.04 (1.30)	4.69 (1.19)	5.52 (1.48)

PF, physical functioning; RP, role limitations due to physical problems; BP, bodily pain; GH, general health perceptions; VT, vitality; SF, social functioning; RE, role limitations due to personal or emotional problems; MH, general mental health; PSQI, sleep score; DASS-DEP, depression score; DASS-ANX, anxiety score; DASS-STRESS, stress score; FSS, fatigue score.

^1^ The eight subscales of the SF-36 range from 0 to 100, where 0 represents the lowest and 100 the highest possible level of HRQoL

^2^ The PSQI global score ranges from 0 to 21, with higher scores indicating poorer sleep quality. A global PSQI score greater than 5 is commonly used to define poor sleep quality

^3^ Each of the three subscales of the DASS-21 ranges from 0 to 21, with higher scores indicating greater symptom severity in the respective domain. The severity cut-offs for each subscale are as follows:

Depression: 0–4 = normal; 5–6 = mild; 7–10 = moderate; 11–13 = severe; ≥ 14 = extremely severe

Anxiety: 0–3 = normal; 4–5 = mild; 6–7 = moderate; 8–9 = severe; ≥ 10 = extremely severe

Stress: 0–7 = normal; 8–9 = mild; 10–12 = moderate; 13–16 = severe; ≥ 17 = extremely severe

^4^ The FSS-9 score ranges from 1 to 7, indicating increasing levels of fatigue severity, with higher scores reflecting greater fatigue. A mean score of 4.0 or above is generally considered indicative of clinically significant fatigue

Table S2 in the Supplementary Material includes the demographic characteristics of the three profiles, along with those of the overall sample for comparison.

### Relationship between clinical parameters at COVID-19 diagnosis and identified profiles

The selected model showed high classification accuracy (entropy = 0.929), indicating clear separation among profiles. Given this high entropy, subsequent analyses examining associations with covariates were conducted using the two-step approach.

**Fig. 2:**
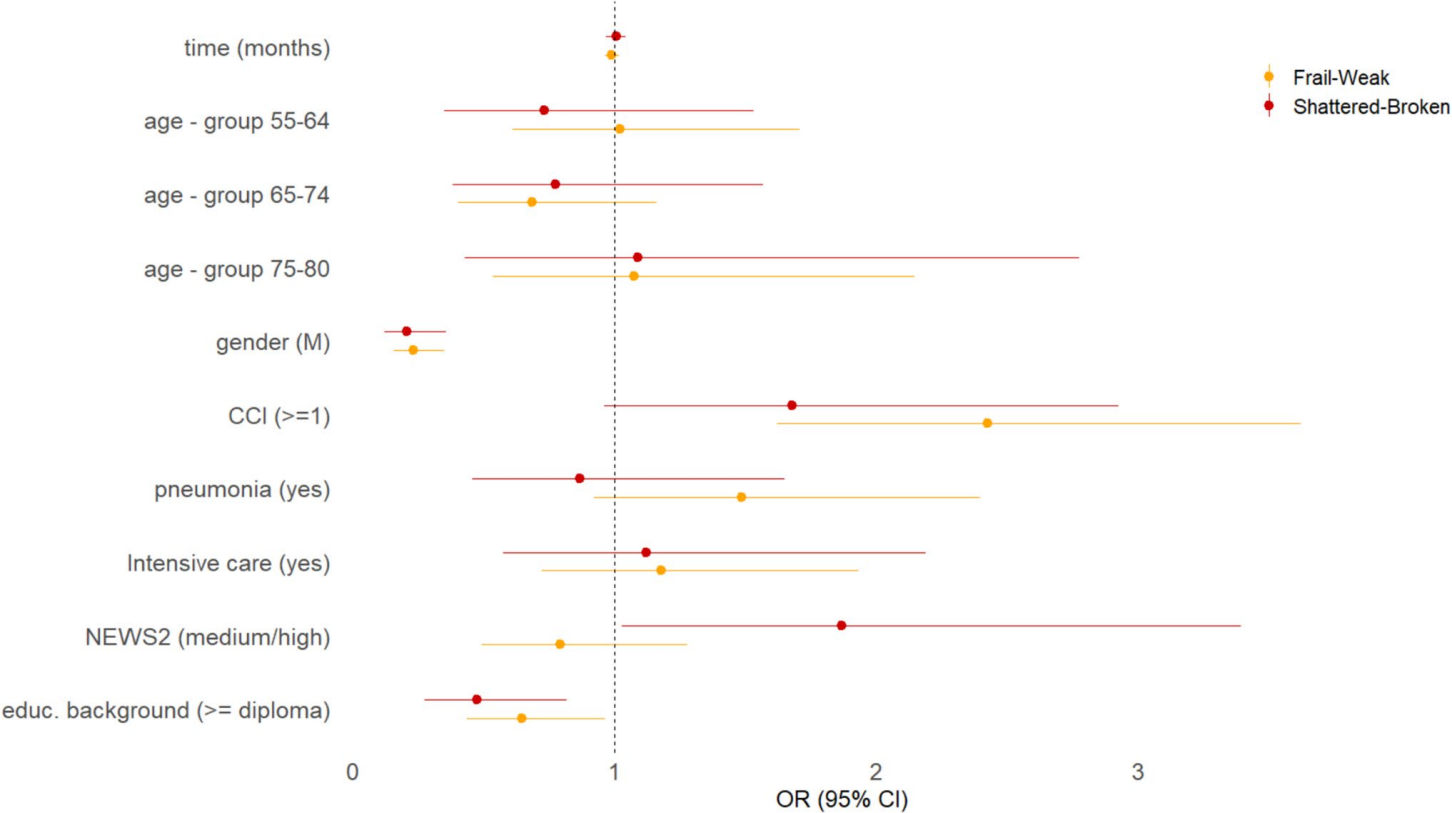
Multinomial logistic regression for profile membership, odds ratios (OR) with 95% CI

Multinomial logistic regression analyses revealed significant associations between profile membership and several demographic and clinical variables (see Fig. 2). The data are presented in comparison to the reference profile *Fit–Vital*. Specifically, male gender and higher educational attainment were both associated with an increased likelihood of belonging to the well-functioning profile (*Fit-Vital*), as opposed to the moderately or severely impaired profiles (*Frail-Weak* and *Shattered-Broken*). Conversely, the presence of one or more comorbidities significantly increased the probability of being classified into the more impaired profiles. Moreover, individuals with a medium or high NEWS2 score were more likely to belong to the *Shattered-Broken* profile than to the *Fit-Vital* group. In contrast, age, time since hospitalisation, history of pneumonia, and admission to intensive care were not significant factors in determining profile membership. Odds ratio and 95% CI estimates are provided in Table S3 of the Supplementary Material.

The data are presented in comparison to the reference profile *Fit–Vital*. The reference group was defined as individuals aged 18 to 54 years, female, with no comorbidities, no history of pneumonia or intensive care admission, with a low NEWS2 score, and an educational level below a high school diploma.

An odds ratio (OR) greater than 1 indicates an increased likelihood of the outcome relative to the reference category, whereas an OR less than 1 indicates a decreased likelihood. The 95% confidence intervals (CIs) reflect the precision of the estimates. An effect is considered statistically significant if the CI does not include 1.

## Discussion

The results of this study highlight the presence of three distinct health-related profiles that emerged a long time after hospitalisation for COVID-19, each characterized by different levels of overall well-being. The *Fit-Vital* profile is associated with good physical and mental conditions; the *Frail-Weak* profile is marked by moderate deterioration, primarily on the physical side, and the *Shattered-Broken* profile shows a marked decline in HRQoL and severely compromised physical and mental health. As hypothesized, two sharply contrasting profiles emerged: one associated with positive physical and mental conditions and the other with severe impairments in HRQoL, sleep quality, depression, anxiety, stress, and fatigue. These findings confirm that COVID-19 infection can lead to highly heterogeneous physical and mental outcomes, some of which are profoundly debilitating in the long term [1, 23, 50, 51]. Positioned between these two extremes is a third, intermediate profile, characterized by moderately impaired physical and mental conditions, with greater vulnerability on the physical rather than psychological dimension. This profile aligns with the considerations in the introduction regarding individual variability in psychological responses to the pandemic. Indeed, previous studies have shown that mental health trajectories during and after the pandemic were not uniform, with many individuals displaying resilience or even positive psychological changes following lifestyle adjustments imposed by the pandemic [8–11]. The emergence of such a profile, showing a more pronounced impact on physical health than on mental health, suggests that, according to self-reported QoL measures, individuals tend to experience a more pronounced perceived impact on physical health than on mental health [7, 52]. When combining the proportions of the two negatively affected profiles (*Frail-Weak* and *Shattered-Broken*), it becomes evident that over 50% of the sample exhibits long-term physical problems, thus underlining the persistent impact of SARS-CoV-2 infection on individuals’ physical health over time. These results are consistent with international evidence reporting a significant proportion of patients experiencing persistent symptoms months after infection, particularly chronic fatigue, muscle pain, and sleep disturbances [53, 54]. No profile was identified in which mental impairments predominated over physical ones. Psychological difficulties appear almost exclusively in conjunction with substantial physical impairment, suggesting that COVID-19 does not result in isolated mental health deterioration, but rather to psychological challenges that are intertwined with individuals’ self-perceived physical well-being [55, 56].

The stratification of post-COVID-19 patients into three distinct profiles, combined with the availability of baseline data collected at hospital admission, enabled the analysis of risk factors associated with membership in each profile. This approach made it possible to identify the predictors of both favourable and unfavourable physical and mental outcomes over time following SARS-CoV-2 infection. In particular, a significant association emerged between certain demographic factors and membership in the profiles characterized by compromised physical and mental outcomes (*Frail-Weak* and *Shattered-Broken*), as compared with the profile exhibiting positive outcomes (*Fit-Vital*). Specifically, female gender and lower educational attainment were found to confer an increased risk of developing physical and mental health problems in the post-COVID-19 period. These associations are well documented in the scientific literature, where women are shown to be more susceptible to persistent symptoms, chronic fatigue, and psychiatric disorders following infection [15, 57–59], and lower educational status is frequently linked to poorer health outcomes, even in pandemic contexts [60, 61].

Clinical factors, including the presence of comorbidities and the severity of the patient’s condition at hospital admission, as assessed by the NEWS-2 score, have likewise been identified as significant predictors of worsening physical and mental health in the post-COVID-19 period. In particular, having more than one comorbidity, measured via the Charlson Comorbidity Index, increases the probability of belonging to the profiles with worse outcomes, and a higher NEWS-2 score is associated with a greater likelihood of falling into the group with severely compromised physical and psychological status (*Shattered-Broken*). Such findings further underscore how the severity of clinical presentation at admission strongly determines long-term physical and mental outcomes after COVID-19 [25, 51, 62, 63]. Contrary to expectations, neither a diagnosis of pneumonia at admission nor intensive care unit (ICU) admission emerged as significant risk factors for the development of physical and mental health complications over time following SARS-CoV-2 infection. This issue remains highly debated in the literature, with contradictory findings reported to date [13, 54, 64–66]. However, the high prevalence of pneumonia (74.5%) and the low proportion of ICU admissions (18.8%) in the present sample may have contributed to an underestimation of this association. This highlights the complexity and variability of post-COVID-19 symptomatology and suggests that initial disease severity and the presence of respiratory complications, such as pneumonia, may influence, but do not necessarily determine, the onset of long-term mental and physical health symptoms.

The delineation of health-related profiles in this study and their impact on overall well-being reflect the most recent evidence in the literature, highlighting the complexity of the systemic response to SARS-CoV-2 infection and emphasizing the need for person-centered strategies within a multidisciplinary, integrated approach for the management of post-COVID-19 sequelae.

While the profiles identified here primarily reflect a continuum of severity across physical and psychological domains rather than qualitatively distinct symptom types, this pattern is consistent with previous research showing high intercorrelations among post-COVID-19 symptoms and the presence of a general multidimensional impairment factor. This suggests that persistent post-COVID-19 conditions may be best conceptualized along a severity spectrum rather than as discrete subtypes.

From a clinical standpoint, recognizing such severity-based profiles can still offer valuable guidance for healthcare providers, supporting the timely identification of individuals at greater overall risk and informing the design of integrated, multidisciplinary rehabilitation strategies that address both physical and psychological needs. In this regard, future research should explicitly incorporate severity-adjusted analyses in the study design to better understand the relative contribution of disease burden, hospitalization factors, and individual vulnerability to post-COVID-19 outcomes. We believe that the identification of differentiated physical and psychological outcome profiles and the analysis of risk factors associated with hospital admission and discharge of COVID-19 patients can thus provide crucial support to clinicians, both in the early recognition of post-COVID-19 conditions and in the planning of targeted and effective therapeutic interventions.

### Strengths and limitations

The main strength of the study lies in its large sample size, which includes patients hospitalised for COVID-19. This not only allows for direct engagement with the disease and its related challenges but also provide access to baseline variables collected at the time of infection. These data allow for the assessment of patients’ physical and mental conditions in relation to various clinical and hospital-related factors and support the identification of distinct patient profiles through Latent Profile Analysis (LPA), providing an objective evaluation of physical and psychological status over time. To the best of our knowledge, this is among the first studies to investigate long term post-COVID-19 conditions using LPA rather than traditional clustering techniques.

However, the cross-sectional design of the study limits its ability to evaluate the impact of COVID-19 at the individual patient level, due to the absence of longitudinal follow-up and baseline HRQoL measurements.

## Conclusions

The main findings of this study suggest the existence of three distinct post-COVID-19 health-related profiles (*Fit-Vital*, *Frail-Weak*, and *Shattered-Broken*) highlighting a wide spectrum of post-COVID-19 conditions, ranging from good to severely compromised physical and mental health. While approximately half of the sample displays favourable physical and psychological functioning, the other half presents with varying degrees of impairment, from mild to severe, warranting serious consideration.

The primary risk factors associated with belonging to the more compromised profiles include female gender and lower educational level at the demographic level, and presence of comorbidities and higher NEWS-2 scores at the clinical level.

These results underscore the importance of acknowledging the long-term impact of COVID-19 and support the use of health-related profiling for early risk stratification and the development of tailored long-term care strategies.

## Supplementary Information

Below is the link to the electronic supplementary material.

Supplementary file1 (DOCX 24 kb)

## Data Availability

The datasets generated during the current study are available from the corresponding author on reasonable request.
