# Supplementary material for: Post-COVID-19 physical and mental quality of life: a latent profile analysis and predictive factors
Source: Qual Life Res. 2026 Mar 13;35(4):96. doi: 10.1007/s11136-026-04206-y (PMC12987790; doi:10.1007/s11136-026-04206-y)
Supplement: Supplementary file 1 — Supplementary file1 (DOCX 24 kb) [file 11136_2026_4206_MOESM1_ESM.docx]

**Supplementary material**

**Post-COVID-19 Physical and Mental Quality of Life: A Latent Profile Analysis and Predictive Factors**

**Quality of Life Research**

Marco Viola, Silvia Testa, Carlotta Sacerdote, Manolis Kogevinas, Eva Pagano, Rosalba Rosato

Corresponding author: Marco Viola
University of Turin, Department of Psychology – Via Verdi 10, 10124 Torino, Italy
Mail: m.viola@unito.it

**Table S1 Internal consistency - McDonald’s omega (ω) - % missing**

| scale | ω | % missing (at least 1 item) |
| --- | --- | --- |
| SF-36 (tot) | 0.97 | 10.8% |
| SF-36 - PF* | 0.96 | 4.2% |
| SF-36 - RP* | 0.92 | 3.5% |
| SF-36 - GH* | 0.84 | 3.8% |
| SF-36 - VT* | 0.90 | 5.0% |
| SF-36 - RE* | 0.86 | 3.0% |
| SF-36 - MH* | 0.91 | 4.5% |
| PSQI | 0.86 | 11.6% |
| DASS-21 (tot) | 0.95 | 7.5% |
| DASS-21 - depression | 0.91 | 3.8% |
| DASS-21 - anxiety | 0.88 | 2.5% |
| DASS-21 - stress | 0.91 | 4.5% |
| FSS-9 | 0.95 | 4.5% |

* McDonald’s ω was computed only for SF-36 subscales including at least three items, as reliability coefficients based on factor models are not appropriate for two-item scales; therefore, ω values were not reported for the Bodily Pain (BP) and Social Functioning (SF) subscales.

**Table S2 Sociodemographic and clinical characteristics of the total sample and latent profiles**

|  |  | **Profiles** | | |
| --- | --- | --- | --- | --- |
|  | **Overall** | **Fit-Vital** | **Frail-Weak** | **Shattered-Broken** |
| N | 601 | 289 | 229 | 83 |
| time - months, mean (sd) | 20.71 (7.57) | 20.93 (7.16) | 20.44 (7.84) | 20.70 (8.23) |
| age, mean (sd) | 61.0 (12.4) | 60.1 (12.7) | 61.8 (11.9) | 61.6 (12.8) |
| gender - female | 226 (37.60%) | 62 (21.45%) | 118 (51.53%) | 46 (55.42%) |
| CCI score, ≥ 1 | 282 (46.92%) | 110 (38.06%) | 131 (57.21%) | 41 (49.40%) |
| pneumonia - yes | 448 (74.54%) | 218 (75.43%) | 172 (75.11%) | 58 (69.88%) |
| Intensive Care Unit - yes | 113 (18.80%) | 56 (19.38%) | 41 (17.90%) | 16 (19.28%) |
| NEWS2 - medium/high | 136 (22.63%) | 67 (23.18%) | 42 (18.34%) | 27 (32.53%) |
| educational background - at least high school diploma | 362 (60.23%) | 194 (67.13%) | 128 (55.90%) | 40 (48.19%) |

**Table S3 Parameters Estimation of the multinomial logistic regression**

| **Variable** | **Frail-Weak vs Fit-Vital** | | **Shattered-Broken vs Fit-Vital** | |
| --- | --- | --- | --- | --- |
|  | **OR [95%CI]** | **p-value** | **OR [95%CI]** | **p-value** |
| (Intercept) | 1.776 [0.832 - 3.791] | 0.138 | 0.868 [0.317 - 2.372] | 0.782 |
| time (months) | 0.991 [0.965 - 1.017] | 0.485 | 1.007 [0.970 - 1.045] | 0.727 |
| age - group 55-64 | 1.023 [0.612 - 1.709] | 0.932 | 0.733 [0.351 - 1.532] | 0.408 |
| age - group 65-74 | 0.686 [0.406 - 1.160] | 0.160 | 0.776 [0.384 - 1.567] | 0.478 |
| age - group 75-80 | 1.074 [0.537 - 2.146] | 0.841 | 1.090 [0.428 - 2.775] | 0.857 |
| gender (M) | **0.234 [0.157 - 0.350]** | **< 0.001** | **0.209 [0.122 - 0.357]** | **< 0.001** |
| CCI score (≥1) | **2.423 [1.622 - 3.620]** | **< 0.001** | 1.677 [0.962 - 2.926] | 0.068 |
| pneumonia (yes) | 1.486 [0.922 - 2.395] | 0.104 | 0.869 [0.457 - 1.651] | 0.668 |
| Intensive Care Unit (yes) | 1.180 [0.722 - 1.930] | 0.509 | 1.122 [0.574 - 2.191] | 0.737 |
| NEWS2 (medium/high) | 0.793 [0.493 - 1.277] | 0.341 | **1.869 [1.030 - 3.391]** | **< 0.05** |
| educational background (at least high school diploma) | **0.648 [0.435 - 0.966]** | **< 0.05** | **0.475 [0.276 - 0.817]** | **< 0.01** |

The data are presented in comparison to the reference profile, "Fit-Vital". The reference group was defined as individuals aged 18 to 54 years, female, with no comorbidities, no history of pneumonia or intensive care admission, with a low NEWS2 score, and an educational level below a high school diploma. Bold font indicates statistical significance
